# Early Discharge, Outpatient Evaluation, and Home Management of Acute Respiratory Failure from COVID-19 in Martinique

**DOI:** 10.4269/ajtmh.22-0629

**Published:** 2023-04-10

**Authors:** Moustapha Agossou, Mathilde Provost, José-Luis Barnay, Jean-Marie Turmel, Elsa Cécilia-Joseph, Dabor Resiere, Moustapha Dramé, KOVIDHOM 972 Working Group

**Affiliations:** 1 Department of Respiratory Medicine, CHU of Martinique, Fort-de-France, Martinique;; 2 Department of Physical Medicine and Rehabilitation, CHU of Martinique, Fort-de-France, Martinique;; 3 Department of Infectious disease, CHU of Martinique, Fort-de-France, Martinique;; 4 Clinical Data Center, CHU of Martinique, Fort-de-France, Martinique;; 5 Medical Intensive Care Unit, CHU of Martinique, Fort-de-France, Martinique;; 6 Department of Clinical Research and Innovation, CHU of Martinique, Fort-de-France, Martinique

## Abstract

A worldwide pandemic of viral infection due to SARS-CoV-2 (and its resultant disease, COVID-19) has been ongoing since 2019. Martinique was affected by a major wave in summer 2021, with saturation of the health system forcing the implementation of home care management. We conducted a retrospective, observational study that included patients treated in the KOVIDHOM 972 program. We included adult patients with SARS-CoV2 hypoxemic pneumonia and requiring 4 L per minute or less of oxygen. In total, 418 were discharged to home with oxygen therapy after hospitalization for SARS-CoV-2 hypoxemic acute pneumonia, and 416 were analyzed. Half (50.2%) were women. Mean age was 58.8 ± 13.0 years. Time from onset of symptoms to hospitalization was 9.1 ± 3.5 days, and average length of stay was 10.5 ± 7.4 days. Maximum oxygen flow during hospitalization was 6.9 ± 4.5 L/min in patients who did not require intensive care. Average oxygen flow at discharge was 1.8 ± 07 L/min. At 30 days after discharge, the readmission rate was 0.5% (95% CI: 0–1.18), and the death rate was 0.5% (95% CI 0–1.18). Our study shows a very low rate of readmission or death in COVID-19 patients discharged to home with oxygen therapy. These results highlight the possibility of safe home care in carefully selected patients. Such programs could be useful in pandemic or wide-scale emergency situations.

## INTRODUCTION

A worldwide pandemic of viral infection due to SARS-CoV-2 (and its resultant disease, COVID-19) has been ongoing since 2019. It has since evolved in epidemic waves in different regions of the world. Managing these waves can be challenging. The pandemic has led to saturation of healthcare infrastructures in both developing and developed countries. The Caribbean is a region where the health system is fragile, although effective inter-Caribbean cooperation has been put in place to deal with the pandemic.^1^

The French West Indies experienced a very intense fourth wave with higher incidence rates than in the regions of metropolitan France. This wave took place from July 1 to October 31, 2021, and was characterized by a total 3,353 hospitalized patients, totaling 4,979 hospital stays in the University Hospital of Martinique, the sole acute admissions hospital in the island. The wave was dominated by the delta variant and characterized by lung involvement. Accordingly, many patients had respiratory failure.

Home oxygen therapy has been used in chronic respiratory failure including COPD for decades, and clear international guidelines exist to guide its implementation.^2^ However, there is a paucity of data regarding the safety of home management programs for patients with SARS-CoV2 pneumonia, particularly for patients requiring oxygen therapy.^3^^,^^4^

Some studies have evaluated the possibility of home care for patients in view of the increased demands on hospital systems caused by the pandemic.^5^^,^^6^ This early discharge strategy has been shown to reduce the length of stay, thereby creating space for other patients.^7^ A study from the Netherlands reported a readmission rate of 8.5% and a mortality rate of 1.3% in a cohort of patients managed from the emergency room, and readmission of 7% in another cohort after hospitalization.^7^

Faced with the epidemic of summer 2021 and an unprecedented overload of the island’s healthcare system, several strategies were devised for effective and efficient care of patients, including proposals for alternatives to hospitalization. Specifically, a program called KOVIDHOM 972 was designed and set up by healthcare professionals from the University Hospital of Martinique to facilitate the early return to home of patients hospitalized for COVID-19, most of whom still needed oxygen despite being clinically stable.

The objective of this study was therefore to evaluate the safety of early discharge in patients SARS-CoV2 pneumonia requiring home oxygen. We hypothesized that a mortality rate of < 1% and a rehospitalization rate < 10% at 30 days would be acceptable in these conditions.

## MATERIALS AND METHODS

We conducted a retrospective, observational study that included patients treated in the KOVIDHOM 972 program between August 1, 2021, and March 31, 2022, who received home oxygen therapy. The study was performed in accordance with the declaration of Helsinki and received the approval of the Institutional Review Board of the University Hospitals of Martinique (no. 2022/0168).

### KOVIDHOM 972 program.

The KOVIDHOM 972 program was designed to enable early discharge to home in patients with acute pneumonia due to SARS-CoV-2 infection who still required oxygen support but were otherwise stable. The aim was to enable safe return to home for these patients while simultaneously freeing up beds at the hospital for new patients.

The inclusion criteria for the KOVIDHOM 972 program are as follows:

- Patients treated at University Hospital of Martinique for acute lung infection due to SARS-CoV2
- At least 12 days elapsed since onset of symptoms
- If the patient still required oxygen at discharge, the flow rate had to be ≤ 4 L/min, and any comorbidities had to be stable
- Patients did not live alone

Patients who were discharged to long-term care or rehabilitation were not eligible for the KOVIDHOM 972 program. Patients who had been in the intensive care unit (ICU) were eligible for the KOVIDHOM 972 program as long as they were being discharged from a conventional ward to home (with or without oxygen therapy) and met the inclusion criteria.

Patients who met the eligibility criteria were included in the KOVIDHOM 972 program and discharged early to home. Once at home, they had regular monitoring by a nurse, two or three times daily. Clinical data and vital signs were communicated to the patient’s general practitioner (GP) by the nurse. A respiratory medicine specialist was on call 24/7 at the hospital so that the community nurses and GPs could contact a respiratory medicine specialist at the hospital at any time if needed. Patients who had home oxygen therapy were followed up with a consultation at the outpatient unit at 1 and 2 weeks, then again at 1, 3, 6, and 12 months. Patients who were still under oxygen therapy at 1 month were followed up again at 2 months, whereas those who were weaned from oxygen at 1 month were only seen again at 3 months.

If the patient’s condition deteriorated at home, the family or nurse called the emergency services for admission via the emergency department. The nurses were provided with an algorithm to taper oxygen requirements down to 1 L/min, but weaning was performed in the hospital’s outpatient unit once patients were able to perform the 6-minute walk test without desaturation and provided they covered a distance of at least 50% of the theoretical distance (determined by age, height, weight, and gender).^8^

### Study population.

In the present study, we included only patients from the KOVIDHOM 972 program who were still under oxygen therapy at the time they were discharged to home. We recorded sociodemographic (age and sex) and clinical characteristics (comorbidities, length of hospital stay, duration of symptoms, degree of lung involvement, maximum oxygen flow required, clinical course after discharge to home, cause of rehospitalization within 30 days after discharge to home). Vital signs (blood pressure, oxygen saturation, respiratory rate, and heart rate) were also collected twice a day by a home nurse.

The primary outcome was rehospitalization rate at 30 days after discharge. Secondary outcomes were mortality at 30 days, unscheduled rehospitalization at 90 days, and the proportion of patients weaned from oxygen at 1 and 2 weeks and at 1 and 3 months.

### Statistical analysis.

Descriptive analysis was performed. Quantitative variables are described as mean ± SD. Categorical variables are presented as number and percentage. Comparisons of means and percentages were performed respectively using Student’s *t* test and χ^2^ test (or Fisher’s exact test as appropriate). Statistical analyses were performed using SAS software version 9.4 (SAS Institute Inc., Cary, NC). Tests were considered as significant for *P* < 0.05.

## RESULTS

**Figure 1.**
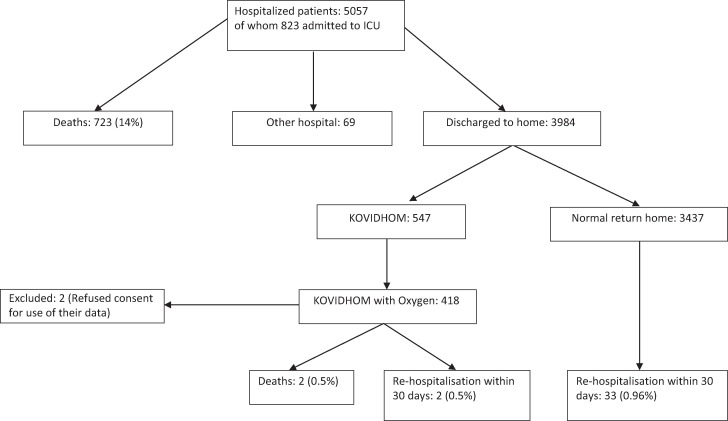
Flowchart of the study population.

We included 547 patients in the KOVIDHOM 972 program from August 1, 2021, to March 30, 2022, of whom 418 were discharged to home under oxygen therapy after hospitalization for SARS-CoV-2 hypoxemic acute pneumonia and 416 included in the study. Figure 1 shows the flowchart of patient outcomes during the study period. Half of the study participants (50%) were women. Mean age was 58.8 ± 13.0 years. The time from onset of symptoms to hospitalization was 9.1 ± 3.5 days, and the average length of hospital stay was 10.5 ± 7.4 days. The maximum oxygen flow during hospitalization was 6.9 ± 4.5 L/min among patients who did not require intensive care. Patients admitted to intensive care accounted for 22.4%, of whom four patients (1%) were intubated. A total of 823 patients were admitted in ICU in the study period.

The average oxygen flow at discharge was 1.8 ± 07 L/min.

In 61% of patients, oxygen was stopped at 7 days, 24% at 15 days, and 12% at 30 days. Only four patients were still on oxygen at 3 months, of whom 3 were stopped. One patient was still receiving oxygen at 6 and 12 months. The characteristics and major comorbidities of the study population are displayed in Table 1.

**Table 1:** Comorbidities and oxygen requirements in COVID survivors enrolled in the KOVIDHOM 972 program in Martinique

Patient characteristics (*N* = 416)	*n*	%
Male sex	207	49.8
Comorbidities		
Arterial hypertension	143	34.4
Diabetes mellitus	88	21.2
Obesity	155	37.3
Obstructive sleep apnea	29	7.0
Heart diseases	19	4.6
Asthma/COPD	18	4.3
Autoimmune diseases	15	3.7
Neoplasia	14	3.4
Kidney diseases	10	2.4
Sickle cell diseases	8	1.9
ICU stays		
ICU hospitalizations	93	22.4
Intubation	4	1.0
Extent of lung involvement		
> 75%	1	0.2
51%–75%	85	20.4
26%–50%	216	52.0
≤ 25%	114	27.4
Oxygen therapy		
Use of high nasal flow oxygen therapy	85	20.4
Maximum oxygen flow (non-ICU patients) (L/min)	6.9 ± 4.5	
Oxygen flow rate at discharge (L/min)	1.8 ± 07	
Oxygen withdrawal		
Day 7	253	61.1
Day 15	100	24.2
Day 30	50	12.1
Day 60	7	1.7
Day 90	4	1.0
Outcome after discharge		
Death within 30 days	2	0.5
Rehospitalization within 30 days	2	0.5
No event	412	99

COPD = chronic obstructive pulmonary disease; ICU = intensive care unit.

Overall, two of 416 patients were readmitted within 30 days, yielding a rehospitalization rate of 0.5% (95% CI: 0–1.18). Mortality was 0.5% (two of 416 patients) at 30 days (95% CI: 0–1.18). There were no additional deaths or unscheduled rehospitalizations at 90 days in this population.

The two cases of rehospitalization concerned a case of cardiac decompensation on day 22 after discharge to home, and one case of pulmonary embolism diagnosed on day 15 after discharge to home. The two patients were treated appropriately and had favorable outcomes.

Both deaths occurred at home. The first death occurred on day 4 after discharge to home; this was a 69-year-old patient with a history of SS sickle cell disease, valvular heart disease, and rhythm disturbances, as well as chronic renal failure without dialysis. The second death occurred on day 30 after discharge to home in an 83-year-old patient already deemed ineligible for intensive care.

## DISCUSSION

This study included 416 patients with COVID-19 pneumonia discharged to home with oxygen therapy. The rehospitalization rate was 0.5% (two patients). The mortality rate was 0.5%. The average length of hospital stay before being discharged to home was 10 days.

We report here one of the largest cohorts to date of patients discharged from hospital to home under oxygen therapy. The rate of rehospitalization observed among these patients is much lower than that found by other authors. Grutters et al. found a rehospitalization rate of 8% among patients receiving home oxygen therapy.^7^ However, in their study, the return to home was earlier, after an average length of stay of 5.1 ± 3.4 days. In the series reported by Banerjee et al., the authors found a rate of 8.5%, after discharge with home oxygen from either emergency or inpatient encounters.^3^ In both studies, discharge was performed as early as possible, and the authors did not apply a minimum of 12 days from symptom onset, as we did in our study.

In the series by Somani et al., 3.8% of patients were readmitted within 14 days^9^ and 7.6% within 30 days in the study by Beiser et al.^10^ These two studies reported registries of patients cared for in-hospital, but without any mention of early discharge and without the need for continued home care.

The main causes of rehospitalization in our study were the onset of respiratory distress in one patient and pulmonary embolism in the other. In our experience, we found that patients could still deteriorate up until 10 days after onset of symptoms, which is why we set the possibility of returning home from the 12th day of symptoms. Because of the frequency of thromboembolic events, we maintained preventive anticoagulant therapy until oxygen withdrawal.

The mortality rate in our series was low, and lower than reported elsewhere. For example, in their study, Banerjee et al reported a mortality rate of 1.3%,^3^ whereas there were no deaths in the series by Grutters et al.^5^

The monitoring and follow-up measures implemented in our study may have contributed to the low rate of rehospitalization observed in our patients discharged to home under oxygen therapy. Nevertheless, we observed two deaths in patients enrolled in the program. These deaths occurred during a period of extreme strain on the hospital, and in this context, the patients could not be admitted to the ICU because of their age and comorbidities. Despite these two deaths, early discharge of patients hospitalized for acute hypoxemic pneumonitis with SARS-CoV2 seems to be feasible, with an acceptable safety profile, provided the patients are well selected.

This is the first program of home management for COVID-19 patients requiring oxygen in the Caribbean region. This program could represent an attractive opportunity for this region to avail of acceptable healthcare alternatives, in view of the fragile healthcare system and the propensity for disastrous natural meteorological phenomena. This program may reduce the length of hospital stay in patients eligible for early discharge under oxygen, thereby freeing up hospital beds for more seriously ill patients. We were careful in the selection of patients, especially in terms of oxygen flow at the time of discharge. In light of this experience, we could expand the criteria for inclusion in the program if there was an epidemic rebound. Early discharge programs such as the KOVIDHOM 972 initiative reported here could be considered in other situations that place hospital under stress, or even in routine care, to reduce the length of stay, provided that appropriate implementation criteria are defined to ensure patient safety.

## CONCLUSION

The KOVIDHOM 972 program of early discharge to home under oxygen therapy in patients with COVID-19 pneumonia was shown to have an acceptable safety profile, with 99% of patients experiencing no events. The length of hospital stay was shorter in those discharged early, thereby increasing the availability of hospital beds for other patients. This program could be applied during other periods of hospital stress, even outside of the pandemic context, such as during natural disasters. Such programs could also be considered in routine care to reduce the length of hospital stay.
